# Monomorphic Epitheliotropic Intestinal T-cell Lymphoma Presenting With Significant Villous Atrophy in the Small Intestine

**DOI:** 10.7759/cureus.79496

**Published:** 2025-02-23

**Authors:** Sotaro Ozaka, Haruhiko Takahashi, Tomoe Hamano, Masahide Fukuda, Kazuhiro Mizukami

**Affiliations:** 1 Department of Gastroenterology, Faculty of Medicine, Oita University, Yufu, JPN

**Keywords:** case report, gastrointestinal endoscopy, intestinal lymphoma, magnifying endoscopy, meitl, monomorphic epitheliotropic intestinal t-cell lymphoma, villous atrophy

## Abstract

Monomorphic epitheliotropic intestinal T-cell lymphoma (MEITL) is a rare and aggressive primary intestinal T-cell lymphoma with a high mortality rate and poor prognosis. Although endoscopy plays a key role in early diagnosis, reports on detailed endoscopic findings are limited. Here, we present a case of MEITL with refractory diarrhea and significant villous atrophy, as observed on endoscopy.

A 67-year-old woman was admitted to our hospital with refractory diarrhea and weight loss. Esophagogastroduodenoscopy revealed microgranular mucosa with significant villous atrophy in the duodenum. Enhanced magnifying endoscopy with narrow band imaging showed flattening and loss pattern of the villi in the transverse part of the duodenum. Colonoscopy also showed significant villous atrophy in the ileum. Biopsy specimens from the duodenum and ileum showed diffuse proliferation of small- to medium-sized atypical lymphoid cells in the lamina propria and intraepithelial lymphocytes. Immunohistochemistry revealed that the cells were positive for CD3, CD8, CD56, and Granzyme B. Diagnosing MEITL, PVPP (sobuzoxane, etoposide, and prednisone) chemotherapy was administered. However, since the patient developed intestinal obstruction after two courses of chemotherapy, it was discontinued. The patient died of intestinal perforation 82 days after diagnosis.

MEITL can cause villous atrophy in the small intestine. Hence, magnifying endoscopy and follow-up histological examination are essential when villous atrophy is observed in patients with refractory diarrhea.

## Introduction

Monomorphic epitheliotropic intestinal T-cell lymphoma (MEITL) is a rare malignant lymphoma of the extranodal lymphoid tissue derived from intestinal T lymphocytes, mainly arising from the small intestine [1]. MEITL is rare accounting for less than 5% of peripheral T-cell lymphomas [1]. It was previously known as enteropathy-associated T-cell lymphoma (EATL) type II, with the term MEITL being newly defined in the 2016 revision of the World Health Organization (WHO) classification of lymphoid neoplasms. It is an aggressive T-cell lymphoma that is known to show rapid progression and has a very poor prognosis due to the poor general condition of the patient at diagnosis or during treatment, and its resistance to chemotherapy [2]. Unlike EATL type I, MEITL shows no definite association with celiac disease and has a higher incidence in Asians [3]. Since MEITL generally presents with nonspecific symptoms such as abdominal pain and diarrhea during the early stages, it underlines the importance of early detection of MEITL by endoscopy and imaging, because early diagnosis of MEITL can help with efforts to improve its prognosis [4,5]. However, since most cases of MEITL involve the small intestine and it is often diagnosed after surgery for intestinal obstruction or perforation, only a few published reports on its endoscopic findings are available [6]. Ishibashi et al. previously reported that edematous or granular mucosa with villous atrophy is an important finding for the early diagnosis of MEITL [7]. It has also been reported that magnifying endoscopy has a higher sensitivity for detecting villous atrophy than standard endoscopy [8].

Here, we present a case of MEITL that presented with refractory diarrhea, in which we were able to observe significant villous atrophy in the duodenum and ileum, an important finding in its early detection, using endoscopy, including magnifying endoscopy.

## Case presentation

A 67-year-old woman visited a previous hospital complaining of persistent diarrhea for 12 months. Colonoscopy revealed villous atrophy at the distal end of the ileum and edematous changes in the ascending colon. The patient was suspected to have celiac disease based on the endoscopic findings and was kept on a gluten-free diet for three months. However, her symptoms did not improve and she lost 5 kg over three months. She was referred to our hospital for further investigation. The patient had no significant personal or family history, except for a history of a right calcaneus fracture. She was 155.7 cm tall, weighed 38.4 kg, and had a body mass index of 15.8. Her blood pressure was 116/72 mmHg, heart rate was 85 bpm with sinus rhythm, and body temperature was 37.5 °C. She had no abdominal tenderness. There was no obvious hepatosplenomegaly or lymphadenopathy. Laboratory tests indicated a hemoglobin level of 9.8 g/dL and an albumin level of 2.45 g/dL. Her serum lactate dehydrogenase (LDH) level was 156 U/L (normal range: 124-222 U/L) and soluble interleukin-2 receptor level was 930 U/mL (normal range: 104-587 U/mL) (Table 1).

**Table 1:** Laboratory data on admission WBC: white blood cell, RBC: red blood cell, MCV: mean corpuscular volume, MCH: mean corpuscular hemoglobin concentration, PT: prothrombin time, APTT: activated partial thromboplastin time, TP: total protein, BUN: blood urea nitrogen, T-bil: total bilirubin, CK: creatinine kinase, AST: aspartate aminotransferase, ALT: alanine aminotransferase, LDH: lactate dehydrogenase, ALP: alkaline phosphatase, γ-GTP: γ-glutamyl transpeptidase, CRP: C-reactive protein, sIL-2R: soluble interleukin-2 receptor, NA: not applicable.

Parameters	Data	Reference	Parameters	Data	Reference	Parameters	Data	Reference
WBC	6690 /μL	3300-8600	TP	5.2 g/dL	6.6-8.1	γ-GTP	11 U/L	9-32
RBC	310×10^4^ /μL	386-492	Albumin	2.4 g/dL	4.1-5.1	Sodium	139 mEq/L	138-145
Hemoglobin	9.8 g/dL	11.6-14.8	BUN	15.2 mg/dL	8-20	Chloride	110 mEq/L	101-108
Hematocrit	29.7%	35.1-44.4	Creatinine	0.46 mg/dL	0.46-0.79	Potassium	5.0 mEq/L	3.6-4.8
MCV	95.8 fL	83.6-98.2	T-bil	0.28 mg/dL	0.4-1.5	CRP	0.09 mg/dL	0-0.14
MCHC	31.6%	27.5-33.2	CK	40 U/L	41-153	sIL-2R	930 U/mL	104-587
Platelet	57.4×10^4^ /μL	15.8-34.8	AST	11 U/L	13-30	HBs antigen	(-)	(-)
NA	NA	NA	ALT	9 U/L	7-23	HCV antibody	(-)	(-)
PT (%)	79%	70-130	LDH	156 U/L	124-222	HTLV-1 antibody	(-)	(-)
APTT	36.7 sec	24-39	ALP	102 U/L	38-113	NA	NA	NA

**Figure 1:**
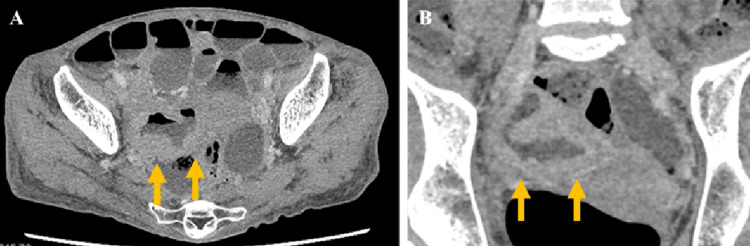
Abdominal computed tomography (CT) images on initial examination Transverse (A) and coronal (B) CT images show thickening of the wall of the ileum (arrow).

Stool culture results were unremarkable. Contrast-enhanced computed tomography (CT) revealed thickening of the ileal walls with ileal dilatation (Figure 1).

**Figure 2:**
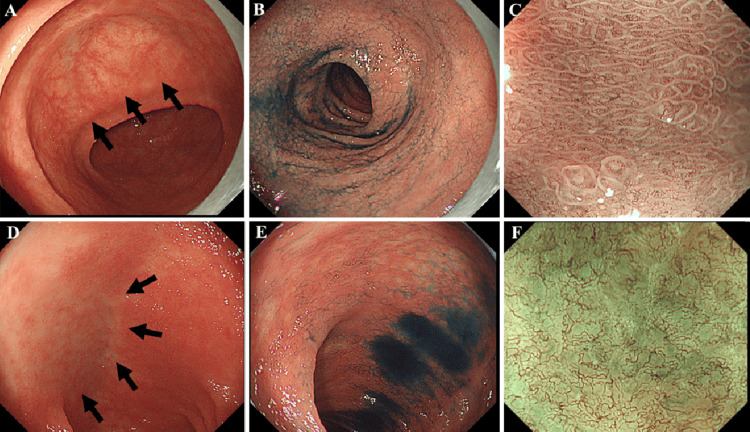
Initial endoscopic examination (A) The transverse part of the duodenum shows visible vascular mucosa (arrow). (B) Indigo carmine spray enhancement reveals microgranular mucosa with a ground cracking pattern. (C) Magnifying endoscopy shows diffusely flattened and blunted villous structures of unequal size, along with complete loss of villous structure. (D) Visible vascular mucosa with villous atrophy is observed in the terminal ileum (arrow). (E) Indigo carmine spray enhancement shows significant villous atrophy. (F) Magnifying endoscopy with narrow band imaging shows villous atrophy with total absence of villi in some areas.

Esophagogastroduodenoscopy showed visible vascular mucosa, resembling “atrophic gastritis”, throughout the duodenum (Figure 2A). Indigo carmine spray enhancement of the transverse part of the duodenum revealed microgranular mucosa with significant villous atrophy (Figure 2B). Magnifying endoscopy showed a diffusely flattened and blunted villous pattern, along with scattered areas of complete loss of villous structure in the transverse part of the duodenum (Figure 2C). Colonoscopy showed visible vascular mucosa with villous atrophy in the terminal ileum (Figure 2D). Indigo carmine spray enhancement showed villous atrophy, while magnifying endoscopy with narrow band imaging (NBI) revealed complete loss of the villous structure (Figures 2E, 2F).

**Figure 3:**
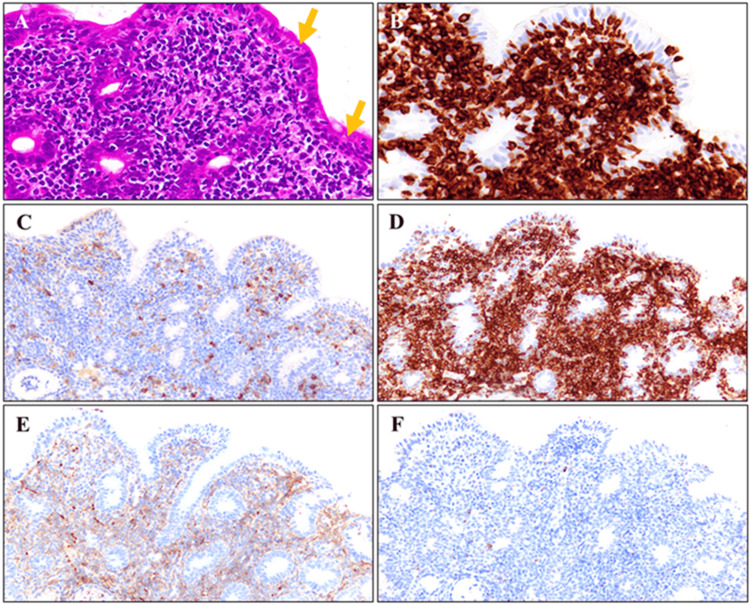
Histological and immunohistochemical analyses (magnification: x400) (A) Hematoxylin-eosin staining shows infiltration of small to medium-sized atypical lymphoid cells in the mucosa. Lymphoid cell infiltration is also observed in the epithelium (arrow). (B-F) The lymphoid cells express CD3 (B), CD4 (C), CD8 (D), CD56 (E), and Granzyme B (F).

There were no abnormal findings in the esophagus, stomach, or colon. Biopsy of the area with villous atrophy in the duodenum and ileum showed diffuse proliferation of small- to medium-sized atypical lymphoid cells in the lamina propria, and intraepithelial lymphocytes. The villi were atrophic, blunted and flattened (Figure 3A). Immunohistochemical analysis revealed that the cells were positive for CD3, CD4, CD8, CD56, and Granzyme B (Figures 3B-3F), but negative for CD5 and CD20.

**Figure 4:**
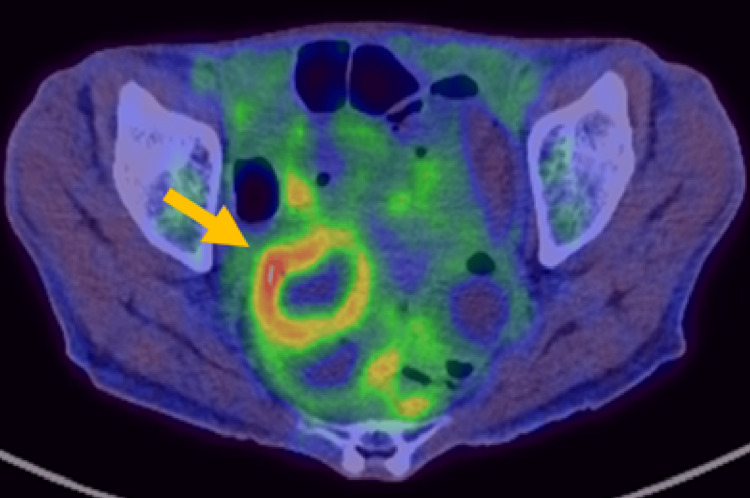
Pre-treatment 18FDG-PET/CT images ^18^FDG-PET/CT shows abnormal uptake in the thickened wall of the ileum (arrow).

Based on these pathological findings, MEITL was suspected. 18FDG-PET/CT that was subsequently performed for identification of systemic lesions showed abnormal uptake in the wall of the ileum, consistent with the findings of contrast-enhanced CT (Figure 4).

There were no abnormal findings on bone marrow aspiration. Based on the features of the tumor and imaging evaluations, we diagnosed MEITL. Thereafter, PVPP (sobuzoxane, etoposide, and prednisone) chemotherapy was administered without dose reduction. However, since the patient developed intestinal obstruction after two courses of chemotherapy, it was discontinued. The patient ultimately died of intestinal perforation 82 days after her diagnosis.

## Discussion

We report a case of MEITL in which we observed significant villous atrophy in the small intestine. Our experience in this case highlights an important clinical point, namely that since MEITL can present with villous atrophy, performing magnifying endoscopy with NBI can aid the diagnosis. Therefore, it is important to suspect MEITL and perform a histological examination when villous atrophy is observed in patients with refractory diarrhea or hypoalbuminemia.

MEITL, previously known as EATL type II, is a primary intestinal T-cell lymphoma that was defined by the 2016 revision of the WHO classification of lymphoid neoplasms [3]. It is a rare disease that accounts for 0.25% of all malignant lymphomas in Japan [9]. EATL was originally categorized into two types, EATL type I and EATL type II. EATL type I is now simply classified as EATL and is common in Western countries. It is closely linked to celiac disease. Tumor cells of EATL type I are typically positive for CD3 and CD30 on immunostaining but negative for CD8 and CD56. MEITL (previously categorized as EATL type II), on the other hand, shows no association with celiac disease and is more common in Asian countries [3]. In this disease, tumor cells are positive for CD3, CD8, and CD56, but negative for CD30 [10]. In the present case, although we initially suspected celiac disease because of the history of refractory diarrhea and endoscopic evidence of villous atrophy, the results of immunostaining led to a diagnosis of MEITL.

Although there are no standardized chemotherapies for MEITL, an anthracycline-based regimen, such as CHOP (cyclophosphamide, doxorubicin, vincristine, and prednisone), is commonly used as the first-line treatment for MEITL [10]. Other regimens include SMILE (dexamethasone, methotrexate, ifosfamide, L-asparaginase, and etoposide) or ICE (ifosfamide, carboplatin, and etoposide) have also been proposed. However, our patient was in poor general condition at the time of diagnosis, and PVPP therapy, which was considered to be well tolerated, was selected. In general, MEITL carries a poor prognosis due to its resistance to chemotherapy and the risk of intestinal perforation or obstruction at diagnosis or during the course of treatment [2]. MEITL has a poor prognosis, with a median survival of 14.8 months and a progression-free survival of 6.9 months. A retrospective analysis reported that Lugano stage I-II1&2 MEITL patients had a significantly better prognosis than those with stage IIE-IV disease (OS 18.8 months vs 4.9 months, p = 0.010) [11]. Moreover, the prognosis of non-perforated cases was significantly better than that of perforated cases in a study of 26 MEITL cases [12]. These studies suggest that early diagnosis and treatment are important for improving the prognosis of MEITL. In fact, cases of MEITL with localized disease with successful chemotherapy and recurrence-free survival for more than five years have been reported [13,14]. However, early diagnosis of MEITL is difficult because most cases of MEITL present with atypical gastrointestinal symptoms in the early stages, and it often occurs in the small intestine [5]. Since the clinical symptoms during the early stages of MEITL are nonspecific, with diarrhea in 56%, abdominal pain in 44%, and weight loss in 44% of cases [15], the importance of endoscopy focusing on minute changes in the gastrointestinal mucosa for early diagnosis has been emphasized in recent years [4].

The endoscopic findings of MEITL have been reported as mucosal edema with diffuse and mosaic mucosal thickening patterns, ulcer, stenosis, and ulcerative-type tumors [15]. A review of 47 cases of MEITL reported ulcers in 33.7%, mucosal edema in 24.1%, a mass in 9.6%, mucosal thickness in 8.4%, and stenosis in 3.6% of patients [16]. Furthermore, Table 2 shows a summary of the 55 cases with confirmed endoscopic findings, including our case.

**Table 2:** Endoscopic findings of MEITL 55 cases MEITL: Monomorphic epitheliotropic intestinal T-cell lymphoma The number of reported cases is 55. When there are multiple lesions or lesions in multiple organs, they are counted in duplicate.

		Lesions	
Site	Esophagus	1 (1.1%)	
	Stomach	4 (4.5%)	
	Duodenum	22 (24.7%)	*
	Jejunum	27 (30.3%)	*
	Ileum	18 (20.2%)	*
	Colon	21 (23.6%)	
Endoscopic finding	Ulcer	34 (44.2%)	
	Mucosal edema	20 (26.0%)	*
	Mass	11 (14.3%)	
	Mucosal thickness	7 (9.1%)	
	Stenosis	5 (6.5%)	
* Findings confirmed in the present case			

In the present case, there were no ulcers or tumors, only microchanges in the mucosa confined to the small intestine, leading to a delay in the diagnosis of MEITL. MEITL is also characterized endoscopically by the presence of diffuse lesions, such as edematous and granular mucosa, in addition to the main lesion. Furthermore, various findings have been reported to describe the changes in the villi, such as white villi, villous atrophy, fusion, and swelling. Ishibashi et al. reported that enteropathy-like lesions in the duodenum and small intestine are important clinicopathologic findings for the early detection of MEITL [7]. Enteropathy-like lesions are histopathologically defined as atrophic villi with intraepithelial lymphocytes (IELs) and are visible endoscopically as edematous or granular mucosa. A review of 24 cases of MEITL in Japan reported the presence of this enteropathy-like lesion in 50-76% of patients [12,17]. In the present case, granular mucosa was observed in the duodenum and ileum, and histopathology showed significant villous atrophy and IEL, which are typical enteropathy-like lesions of MEITL. In previous reports, enteropathy-like lesions were found in more than half of MEITL cases, suggesting that attention to microscopic mucosal findings, including villous atrophy, is essential for the early diagnosis of MEITL.

Magnifying endoscopy with NBI has been reported to be more sensitive for detecting villous atrophy than standard endoscopy [8]. In a recent meta-analysis, NBI had a sensitivity of 93% (95% CI: 81-98%) and specificity of 95% (95% CI: 92-98%) for detecting villous atrophy [18]. Furthermore, combining NBI with magnifying endoscopy improves diagnostic accuracy [19]. Therefore, although the use of magnifying endoscopy with NBI might increase the likelihood of early detection of MEITL, few studies have evaluated its role in the diagnosis of MEITL. In the present case, diffusely flattened and blunted villous structures of unequal size and complete loss of villous structure were observed on magnifying endoscopy. These findings resembled the pattern of villous atrophy seen in celiac disease. De Luca L et al. classify the magnifying endoscopy with NBI findings of celiac disease into abnormal villous patterns and absent villous patterns. The former finding is represented as “a low-density of villi that appear irregular, disoriented, and with an initial pattern of surface destruction”, and the latter finding as “marked villous atrophy, with a flat surface, and with a total absence of villi” [20]. Given that celiac disease is characterized by histopathologic villous atrophy with IELs, the magnifying endoscopic findings of the villous structures seen in MEITL are probably similar to those of celiac disease, making a distinction between the two conditions difficult. Therefore, follow-up histological evaluation is essential. Due to the rarity of the disease, the usefulness of magnifying endoscopy in the diagnosis of MEITL has not been established, and further accumulation of cases is desirable. Lastly, we believe that aggressive endoscopic diagnosis using small bowel endoscopy or magnifying endoscopy and early introduction of chemotherapy are important to improve the prognosis of MEITL.

## Conclusions

In conclusion, we described here a case of MEITL, in which we observed marked villous atrophy in the duodenum and ileum. Because MEITL generally presents with nonspecific symptoms such as abdominal pain and diarrhea during the early stages, it underlines the importance of early detection of MEITL by endoscopy and imaging.

Since villous atrophy is an important endoscopic finding for the early diagnosis of MEITL, it is important to suspect MEITL and perform a histological examination when villous atrophy is observed in patients with refractory diarrhea or hypoalbuminemia.
